# Emerging Utility of Applied Magnetic Resonance Imaging in the Management of Traumatic Brain Injury

**DOI:** 10.3390/medsci9010010

**Published:** 2021-02-14

**Authors:** Jeffrey Nadel, Joseph Scott McNally, Anthony DiGiorgio, Ramesh Grandhi

**Affiliations:** 1 Clinical Neurosciences Center, Department of Neurosurgery, University of Utah, Salt Lake City, UT 84132, USA; jeff.nadel@hsc.utah.edu; 2 Department of Radiology and Imaging Sciences, University of Utah, Salt Lake City, UT 84132, USA; scott.mcnally@hsc.utah.edu; 3 Department of Neurological Surgery, University of California at San Francisco, San Francisco, CA 94143, USA; anthony.digiorgio@ucsf.edu

**Keywords:** traumatic brain injury, magnetic resonance imaging, functional MRI, diffusion tensor imaging, MR perfusion, MR spectroscopy

## Abstract

Traumatic brain injury (TBI) is a widespread and expensive problem globally. The standard diagnostic workup for new TBI includes obtaining a noncontrast computed tomography image of the head, which provides quick information on operative pathologies. However, given the limited sensitivity of computed tomography for identifying subtle but meaningful changes in the brain, magnetic resonance imaging (MRI) has shown better utility for ongoing management and prognostication after TBI. In recent years, advanced applications of MRI have been further studied and are being implemented as clinical tools to help guide care. These include functional MRI, diffusion tensor imaging, MR perfusion, and MR spectroscopy. In this review, we discuss the scientific basis of each of the above techniques, the literature supporting their use in TBI, and how they may be clinically implemented to improve the care of TBI patients.

## 1. Traumatic Brain Injury: Epidemiology and Standard Work-Up

Traumatic brain injury (TBI) is a widespread and costly problem around the world. It is estimated that its global incidence exceeds 27 million new cases annually, which is likely an underestimate given the difficulty in obtaining accurate and timely data [1]. The high incidence not only leads to considerable strain on global health systems in managing the acute treatment and long-term sequalae of TBI, but it also burdens local, national, and international economies via lost worker productivity and the cost of care. The causes of TBI vary widely based on geography and demographics, but common causes include motor vehicle collisions, bicycle crashes, mechanical falls, sports-related injuries, and violence [2]. Understandably, given the preventable nature of many of these injuries, public health interventions have increasingly focused on targeting upstream factors that may lead to a reduction in TBI burden.

In this review, we consider the definition of TBI to be any disruption in consciousness, motor function, sensory function, autonomic function, or ordinary brain function, whether transient or permanent, that is the result of a traumatic injury to the head. Clinically, the severity of TBI is graded using the Glasgow Coma Scale (GCS), where mild TBI is defined as GCS > 13, moderate TBI as GCS 9–12, and severe TBI as GCS 3–8 [3]. When a patient presents with suspected TBI, work-up usually focuses on a obtaining a swift neurological examination and, if indicated, cranial imaging. In the United States, most centers follow the American College of Radiology Appropriateness Criteria for determining whether a patient ought to receive a computed tomography (CT) scan of the head in the acute setting. These guidelines recommend obtaining a noncontrast CT of the head in any patient who presents with a moderate or severe TBI (GCS < 12) to rule out an operable injury, such as an acute epidural hematoma or subdural hematoma [4]. Furthermore, CT angiography (CTA) may be used to rule out any vascular injury after TBI, especially in the setting of skull base fractures [4,5,6]. Although CT is sensitive for identifying large intracranial pathologies, more subtle changes from TBI may not be visible. CT is limited in allowing clinicians to prognosticate on the ultimate functional outcome for a TBI patient. Moreover, and especially in pediatric patients, exposure to ionizing radiation with CT adds risk [7]. This may outweigh benefits of using repeated CT studies in close temporal proximity. For this reason, there has been significant interest in exploring the role of more advanced and emerging imaging techniques in the work-up and surveillance of patients with TBI.

In this review, we focus on the use of magnetic resonance imaging (MRI) and its advanced applications in the management of patients with TBI. Specifically, we will discuss the applications and limitations of general MRI, functional MRI (fMRI), diffusion tensor imaging (DTI), MR perfusion (MRP), and MR spectroscopy (MRS) in TBI. We briefly present alternative non-MRI modalities as well. Our aim is to inform neurosurgeons, neurocritical care physicians, neurologists, emergency physicians, and general physicians who treat TBI patients about newer technologies to care for this patient population.

## 2. MRI in TBI

MRI has been increasingly identified as a powerful tool in the prognostication of recovery from TBI. At its core, standard clinical MRI involves subjecting the patient to a 1.5 T or 3.0 T main magnetic field (B0), gradient coils providing small magnetic field variation for spatial localization, and radiofrequency proton excitation with subsequent decay, signal detection, and postprocessing. By varying the frequency and timing of the magnetic pulses, it is possible to localize the changes in radiofrequency signals to a particular place within the body. Additionally, the rate at which different protons return to their resting state helps distinguish separate tissues. Postprocessing of these signals allows for the construction of images that can be used in the diagnosis and monitoring treatment of disease [8,9].

**Figure 1:**
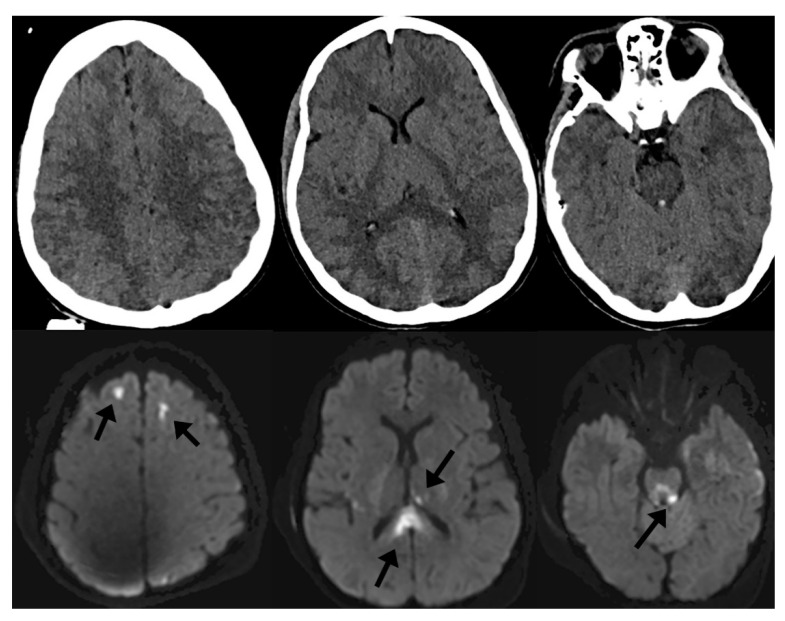
Noncontrast computed tomography (CT) of the head (top row) and diffusion-weighted imaging (DWI) of the brain (bottom row) of the same patient after traumatic brain injury (TBI). Imaging markers of diffuse axonal injury (DAI) include microhemorrhages on susceptibility-weighted imaging (SWI) as well as focal areas of cytotoxic edema on DWI (arrows) that are not visible on CT.

In the setting of TBI, specific conventional MRI sequences such as diffusion-weighted imaging (DWI) and susceptibility-weighted imaging (SWI) tend to be helpful in understanding the clinical implications of injury. In moderate-to-severe TBI, it is common to identify small, scattered microhemorrhages within the brain parenchyma after injury. These microhemorrhages, when localized to the gray–white junctions or white matter tracts, are associated with diffuse axonal injury (DAI). DAI has been extensively studied in the clinical—and less so the pathological—literature and is a negative prognostic factor in recovery from TBI [5,10,11,12,13,14,15,16,17,18]. Often, microhemorrhages related to shear injury are invisible on noncontrast CT because of limited tissue contrast with CT as well as dose reduction limitations in humans and associated noise. However, DWI and SWI sequences of MRI are sensitive enough to detect such small areas of cytotoxic edema and microhemorrhage and are high-sensitivity markers of DAI. Figure 1 demonstrates this phenomenon, where punctate microhemorrhages are difficult to visualize on head CT but are readily apparent on SWI. For this reason, MRI has become an important adjunctive imaging modality in cranial neurotrauma as a means to prognosticate on the expected cognitive and behavioral outcomes of patients [19].

In recent years, emerging technologies have enabled the development of more-advanced applications of MRI. Such diagnostic tools may be useful adjuncts in the early and late post-trauma periods to enable clinicians to better understand the impact of a TBI for an individual patient and augment their treatment accordingly [19]. The remainder of this review will focus on the use of advanced MRI applications in the setting of TBI.

## 3. Functional MRI (fMRI)

fMRI is an application of MR technology whereby clinicians can differentially identify areas of brain activation during specific tasks or in the resting state. The basis of fMRI relies on what is known as the blood oxygenation level–dependent effect [8]. This effect comprises two primary assumptions: (1) as oxygenated hemoglobin transitions to deoxyhemoglobin or vice versa, there is a small but detectable change in the magnetic properties of the heme related to iron oxidation status; and (2) increased neuronal activation in a particular brain region has an associated increase in local cerebral blood flow and oxygen extraction. Taken together, as certain regions of the brain are increasingly activated during a task, there is a local increase in cerebral blood flow and oxygenated hemoglobin transitions to deoxyhemoglobin at increased rates. This produces a subtle change in the magnetic signals in that region, and signal averages over long imaging times can be detected on MRI [20]. A similar but distinct technique, known as resting-state fMRI, is a method of fMRI that employs regional resting-state conditions during rest or in the absence of a particular task. Resting-state conditions are similarly quantified using regional blood flow changes, and alterations in the resting state may identify TBI-related pathology [20].

fMRI has shown promise in TBI. In both the early and late post-trauma periods, many patients remain in states of altered consciousness [21]. Given that one of the fundamental goals in the care of patients with moderate and severe TBI is to return the patients’ state of altered consciousness to its pretrauma baseline, the ability to predict whether and how quickly a patient may recover from their injury has significant implications in TBI prognostication, resource utilization, and guiding families in shared decision-making. However, there is a paucity of clinical and preclinical evidence supporting these efforts [22,23].

Great effort has gone into understanding the differences in functional connectivity of the brain during wakefulness, chemically induced sleep, sleep, and pathologic alterations of consciousness [24,25,26,27]. In 2006, a landmark study demonstrated that patients may display a phenotype known as cognitive–motor dissociation—a type of covert consciousness whereby a patient is cognizant of their surroundings but unable to outwardly demonstrate it [28]. Active investigation has since commenced in identifying the pathophysiology of this phenotype and using fMRI and electroencephalography clinically to predict which patients may experience cognitive–motor dissociation [29,30,31,32]. Trials aimed at using fMRI to identify early return of consciousness in patients after severe TBI have begun [33]. Further research into the use of fMRI to track the return of consciousness in patients after TBI may eventually allow clinicians to use fMRI as a means of prognosticating whether and how quickly a patient may recover.

## 4. Diffusion Tensor Imaging (DTI)

DTI is an application of DWI that has powerful applications in understanding the functional connectivity of the brain. As with DWI, in the acquisition of DTI images, the diffusion of water molecules is quantified within the tissue slab. However, specifically with DTI, multiple parameters are acquired, including the rate at which water molecules diffuse in the tissues as well as the direction of that diffusion. These parameters are acquired for each voxel of the MR image. From those data, specific measures are calculated to describe water diffusion in tissues, including anisotropy and diffusivity [19,34,35]. Broadly, these values are thought to correlate with the biological integrity of the brain’s white matter, as water will more readily diffuse down intact tracts. Higher anisotropy and lower diffusivity are correlated with greater white matter integrity. For this reason, these values have clinical implications [36]. Additionally, because the data collected include a directional component to the diffusion, postprocessing allows for the identification and tracing of specific axonal tracts within brain tissue. These fiber tract renderings are used clinically in neurosurgical operative planning.

The literature is rich with studies that demonstrate that decreased anisotropy and increased diffusivity are common in those with TBI compared with normal controls [36,37,38,39,40,41,42,43]. This pattern is thought to be the MR representation of a trauma-induced disturbance in the microstructure of the axonal tissue [36]. Furthermore, certain local brain regions, including the frontal lobes, the corpus callosum, the centrum semiovale, and the internal capsule, may be more susceptible to these changes after TBI than others [42,44,45,46,47,48]. Although detecting these changes may be helpful in the diagnosis of CT-negative mild TBI, it is less clinically relevant for those in whom TBI has already been confirmed. However, if DTI characteristics could be associated with downstream TBI outcomes, DTI would be of great clinical utility.

Currently, the application of DTI to TBI patients at the individual level is limited because of the lack of normative population-scale data on DTI parameters, as well as the paucity of prospective data on the association between DTI parameters and eventual outcomes. However, recent studies suggest that it may be possible to use DTI parameters in TBI outcome prediction. In a meta-analysis evaluating these studies, higher anisotropy and lower diffusivity were associated with improved downstream cognition, specifically memory and attention [49]. The TRACK-TBI investigators demonstrated that reductions in anisotropy in at least one region of interest within the brain were significantly associated with unfavorable 3- and 6-month outcomes [50]. Another study of former National Football League players showed a subtle but detectable association between the presence of regions of lower anisotropy on DTI and downstream cognitive impairments and depression after recurrent head trauma [44]. Each of these studies is limited by the lack of normative DTI data for comparison, but they together suggest that, as further research in this area develops, DTI may be a useful tool in TBI outcome prediction.

## 5. MR Perfusion (MRP)

MRP is an advanced MR technique that is commonly used to determine and track intracerebral blood flow dynamics. Dynamic susceptibility contrast (DSC) imaging is a perfusion technique in which gadolinium contrast is administered and the decrease in T2 * signal (susceptibility) is quantified as the contrast bolus passes through the brain [51]. The most commonly calculated parameters from DSC include cerebral blood volume, cerebral blood flow, mean transit time, and time to peak of the contrast bolus through the tissues. A similar perfusion technique, known as dynamic contrast-enhanced (DCE) imaging, relies on the T1-shortening effects of the gadolinium and, as such, signal increases as the bolus passes through the tissue. From these regional signal changes, it is possible to calculate parameters that include the rate of perfusion by understanding the fractional volume of gadolinium in the extravascular–extracellular space compared with the fractional volume of gadolinium in the plasma [52]. These differences in T1 and T2 * signal are due to the T1 and T2 shortening effects of gadolinium, resulting in high T1 and low T2 or T2 * signal, respectively. A third technique, known as arterial spin labelling, is a unique perfusion sequence that does not require intravenous contrast administration. It harnesses the ability of the MRI to selectively label inflowing arterial blood and monitor tissue perfusion. In so doing, the protons in flowing arterial blood act as endogenous contrast to calculate bolus parameters such as cerebral blood flow [53,54].

Perfusion-based techniques offer important information in the setting of TBI. Secondary brain injury results from poor perfusion of brain tissue. Poor brain perfusion may occur for a number of reasons, including hypotension, hypovolemia, local mass effect from residual blood products or cerebral edema, or elevated intracranial pressure. Such hypoperfusion leads to further cellular brain injury due to breakdown of the blood–brain barrier, cellular excitotoxicity, neuroinflammation, free-radical generation, and more [55,56,57,58]. Ongoing assessment of brain perfusion might assist clinicians with early and dynamic identification of regional or global cerebral hypoperfusion, so that early interventions can minimize further secondary injury.

Given the ease of use and widespread availability of CT perfusion, MRP has yet to gain significant traction in the day-to-day management of TBI patients; however, because of the power of MRP to detect more-subtle changes in the brain, it has been studied in TBI patients as a means to predict whether and what type of cognitive deficits a TBI patient may be expected to have. It may also aid with understanding the impact of injury on vascular and blood–brain barrier integrity [59]. For example, one study employed DSC to evaluate perfusion deficits in military members after TBI. The authors demonstrated that early perfusion deficits within the anterior cingulate cortex and cerebellum were associated with future neurobehavioral impairment and difficulty with coordination and reaction time testing [60]. Unlike DSC, DCE has not been extensively studied in human TBI populations; however, in preclinical models of acquired TBI, the calculated parameters have been shown to describe blood–brain barrier integrity and cerebral vascular permeability and to correlate with 30-day functional outcome [43,61]. Presumably, as DCE is further investigated for use in TBI, it may provide early pathophysiologic information regarding the severity of TBI and how it may affect prognosis. Finally, across the spectrum of TBI severity, arterial spin labelling is another MRP technique with sensitivity in detecting decreased thalamic, posterior cingulate, and frontal cortical perfusion in TBI patients compared with controls [62,63]. The clinical relevance of such decreased perfusion remains unknown to date; however, given that many patients with moderate and severe TBI are also polytrauma patients with multiple impacted organ systems, the ability to assess brain perfusion without requiring contrast administration is advantageous in such an acutely ill population and so these techniques warrant further study.

## 6. MR Spectroscopy (MRS)

MRS is an imaging technique that enables brain tissue to be analyzed noninvasively for the presence and concentration of specific biochemical metabolites. The underpinnings of MRS rest in the principle that the atoms of different molecules have different proton and electron configurations. This causes them to react differently to the presence of a magnetic field. By varying the frequencies of that magnetic field, it is possible to detect separate signals from different metabolites and therefore differentiate the metabolic composition of the tissue [64,65,66]. Specifically, most centers rely on 1H-MR spectroscopy, which uses the signal from protons (1H) to assess the composition of metabolites that are commonly found in brain tissue. Common biomarkers quantified in the brain include choline (a substrate for cellular membrane synthesis), N-acetylaspartate (NAA, a neuronal metabolite), myo-inositol (a glial metabolite), mobile lipids that originate from small isotopically tumbling microdomains embedded within the plasma membrane or stored in cytoplasmic intracellular lipid droplets (increased with higher levels of apoptosis and necrosis), neurotransmitters such as glutamate and GABA, antioxidants such as glutathione, biochemical byproducts such as lactate, and more [64,65,67]. Previously, 31P-MRS was commonly used because it allowed for labeling of substrates of the tricarboxylic acid cycle (TCA) cycle, including pyruvate and ATP [68]. Early in its history, use of MRS surged to aid in the diagnosis of brain neoplasms, demyelinating conditions, hypoxic–ischemic encephalopathy, and inherited metabolic disorders, and more recently it has been increasingly used in the diagnosis and management of TBI.

In TBI, aside from assessing the burden of DAI-associated microhemorrhages in the brain, it has been difficult to numerically quantify the impact of injury on brain tissue; however, MRS has allowed for biomarker assessment of injury severity. NAA is the most commonly studied metabolite, and decreased concentrations detected in tissue may represent neuronal or axonal damage [69,70]. Similarly, 31P-MRS has been used to detect brain alkalosis from TCA cycle changes in acute TBI. Although clearly academically interesting, the clinical applications of MRS rest in the ability to correlate metabolic biomarkers with ultimate outcomes.

One study demonstrated that NAA, myo-inositol, and neurotransmitter concentrations were correlated with cognitive outcomes after pediatric TBI [71]. 31P-MRS has been used to study brain alkalosis after TBI, and such changes were associated with an unfavorable outcome [68]. In pediatric nonaccidental trauma patients, NAA:creatine and NAA:choline ratios from 1H-MR spectra were significantly associated with poor neurologic outcomes [72]. Another prospective study in pediatric TBI patients showed that NAA concentrations in subcortical brain regions in the early post-trauma period accurately predicted long-term cognitive outcomes [73].

There are, however, limitations to this technique. One study that was recently published was unable to detect differences in metabolites between patients with mild TBI and normative controls [74]. This suggests that MRS may not have the sensitivity to detect subtle metabolic changes from milder injuries and may have greater utility for more severe TBI. MRS may prove to be a useful tool to aid in the early prognostication of TBI patients, although further normative data and study will be necessary before widespread adoption occurs.

## 7. Non-MRI Modalities for Imaging in TBI

Beyond MRI, other radiographic modalities have been studied for use in the work up and management of TBI. These include CTA, transcranial Dopplers (TCDs), positron emission tomography (PET), single-photon emission computed tomography (SPECT), electrophysiologic techniques such as magnetoencephalography (MEG) and electroencephalography (EEG), and near-infrared spectroscopy (NIRS). These modalities may be useful when there is a need for assessment of direct vascular injury or ongoing evaluation of traumatic vasospasm (CTA), to study brain metabolism and thereby assess tissue damage (PET and SPECT), to determine the severity of TBI and assess the physiologic function of the brain (EEG and MEG), or to identify locations of increased brain activity and potentially to reliably monitor brain tissue oxygen content noninvasively (NIRS). These applications are beyond the scope of this MRI-specific review, but offer many important options for assessing the brain after TBI.

## 8. Conclusions

Rapid technological advancement has allowed for the development of remarkable new imaging tools to aid in the diagnostic work-up, management, and prognostication of TBI patients. The standard workup for TBI at this time uses noncontrast head CT as a screening tool for large operative pathologies and, where indicated, contrast-enhanced CTA for suspected vascular injury. With the widespread adoption of conventional MRI as standard of care in the management of TBI, we will likely see greater use of advanced MR-associated technologies such as fMRI, DTI, MRP, and MRS. More than ever before, the treatment of TBI will begin to involve assessment of trauma-induced changes to the brain’s microarchitecture as well as incorporate the study of its post-trauma cellular metabolism. With time and further study, these technologies will eventually afford us a greater ability to prognosticate on eventual outcome and tailor our therapies to a patient’s physiology at a specific point in time.

## Data Availability

Not applicable.
